# Saving Fuel

**Published:** 1875-02

**Authors:** 


### SAVING FUEL.

IF a plate of iron is placed in the bottom of a common grate, and a coal fire is kindled over it in the ordinary way, about one-fourth of fuel is claimed to be saved, with an increase of warmth. Keep the plate clear of ashes.

A large saving of furnace fuel may be made by having a wide and shallow pot, as in the pattern of Dixon & Sons, of Philadelphia. This gives a broad surface of coal, giving out a proportionately large amount of heat in a shorter time; hence the coal is warmed and kindles more rapidly. It is a great waste of heat to use large lumps of coal. If the size used for ranges were burned in furnaces, putting on a little at a time and frequently, a steadier and quicker heat, with less fuel, would be the result—one-sixth less, at least. The more you stir a coal fire, the more it won't burn, because the ashes which contain the warmth necessary to heat the coal up to the kindling point are let down into the ash-pan.

The time to replenish a coal fire is when the surface is red hot. Do not wait until the coals are of a dead heat, or are whitened with ashes.

When you look at your stove or grate in the morning, you may save your kindling many times if there are some hot coals, not by riddling out the ashes, but put on a quart or two of small coal; open the draft or apply the blower, and when the small coal is red hot, put on larger; and when that is fairly kindled, riddle out the ashes.

A bit of coal must absorb a certain amount of heat before it kindles—hence, a small lump of coal gets hot sooner than a arge one, and is therefore more easily and the sooner kindled. In kindling a fire, concentrate the flame as much as possible on the smallest surface of coal.

